# Characterization of the Variables Related to Reports of Death Due to Canine Bites in Scientific Articles during the Years 2013–2017: A Systematic Review

**DOI:** 10.3390/ani11092654

**Published:** 2021-09-09

**Authors:** Carmen Luz Barrios, Valentina Aguirre-Olea, Carlos Bustos-López, Sandra Pérez-Vergara, Sandra Claros-Alegría

**Affiliations:** 1 Escuela de Medicina Veterinaria, Facultad de Ciencias, Universidad Mayor, Camino La Pirámide 5750, Huechuraba 8580745, Chile; sandra.perez@mayor.cl (S.P.-V.); sandra.claros@mayor.cl (S.C.-A.); 2 Departamento Disciplinario de Ciencias de la Documentación, Universidad de Playa Ancha, Valparaíso 2360072, Chile; val.aguirre.olea@gmail.com; 3 Departamento de Ciencias Básicas, Facultad de Ciencias, Universidad Santo Tomás, Av. Ejército Libertador 146, Santiago 8320000, Chile; cbustoslopez@santotomas.cl

**Keywords:** dog, canis familiaris, forensic veterinary medicine, potentially dangerous dogs

## Abstract

**Simple Summary:**

Dog bites are a major public health problem throughout the world. The main consequences for human health include physical and psychological injuries of varying proportions, secondary infections, sequelae, risk of transmission of zoonoses and surgery, among others, which entail costs for the health care system and those affected. The objective of this review was to search and analyze the indexed scientific literature on canine bites resulting in death, published during the period 2013–2017. The results show that most of the articles on bite accidents resulting in death analyzed in this study have details about the victims, their injuries and the treatment received by the victim. These results reflect that most of these accidents are suffered by adults, in public spaces and by only one animal. The analysis of these antecedents, as well as others incorporated in this review, will be of great help to fully understand the incidents of dog bites resulting in death published in the scientific literature.

**Abstract:**

Canine bites are an important public health problem, with consequences such as physical injuries, psychological trauma, transmission of zoonoses, infections and they can even cause death. To avoid deaths caused by this type of bite, multiple factors related to this issue must be considered. The objective of this review was to search and analyze the indexed scientific literature on canine bites resulting in death, published during the period 2013–2017. A search was carried out in various databases of indexed literature, in Spanish and English. After selecting and excluding items using PRISMA, they were classified according to SIGN guidelines to filter out the level of evidence and potential biases. Thirty-three scientific articles were retrieved and analyzed. In most of these, victims between 50 and 64 years of age (28.6%, 8/28) were registered. Additionally, in most of the articles, only one animal participated (80%, 16/20). The highest number of events occurred in public spaces (58.5%, 7/12). In conclusion, most of the scientific articles that report incidents of dog bites resulting in death, provide details about the victims, their injuries and the treatment received; however, few articles provide background information on the context of the attack and the biting animal.

## 1. Introduction

Dog bites are a major public health problem in children and adults worldwide, with dogs being responsible for 76% to 94% of animal bites to people. These incidents affect the physical and psychological integrity of the victims, generate economic costs associated with medical care and reconstructive procedures and also constitute a zoonotic risk [1,2].

As a result of a canine bite injury, there is a high probability of transmission of pathogenic microorganisms, mainly to immunocompromised individuals, which can contract infections by *Streptococcus canis, Pasteurella* spp., *Prevotella* spp. [3] and *Capnocytophaga canimorsus*, the latter being capable of causing meningitis in immunocompromised patients [4]. These injuries can also transmit the rabies virus [5].

Injuries caused by dog bites can sometimes be fatal. There are predisposing factors associated with human deaths that are described in case reports, for example splenectomized people whose death is related to septic shock due to *Capnocytophaga canimorsus* [6]. Moreover, cognitive impairment or other mental or physical disability, alcohol or drug abuse and being homeless have been reported as predisposing factors [7,8], since these subjects do not have the physical ability to defend themselves or ask for help when attacked by a dog [9]. In addition, there are other conditions, such as cultural beliefs, lack of information or cost of treatment, that are related to deaths from the rabies virus [5,10].

Incidents resulting in death in children, with the highest incidence in children under one year of age [11,12], are mainly associated with exsanguination and air embolism, with fatal injuries predominantly located in the head and neck [13]. In the context of this type of attack, the participation of at least two dogs is common. Victims are suddenly bitten by dogs they own or know, and the child’s behavior (running and yelling) triggers predatory behavior in the dog [13]. Additionally, children are often not able to read the warning body language displayed by dogs prior to an attack [14] Their height would explain the predominant location of head and neck injuries. It is important to consider that leaving a dog with a child unsupervised can facilitate the presentation of the attack, leading to the aforementioned injuries.

Factors related to the dog that can lead to a fatal attack on humans are a possible genetic predisposition to aggressiveness, since in a study by Våge et al., an increase in the expression of the *UBE2V2* and *ZNF227* gene was found to be associated with the aggression phenotype [15]. Other authors mentioned that the heritability of aggressiveness is low in the canine population in general, but that despite this low heritability, it may be higher in some individuals [16]; thus, through selective breeding, we could reduce aggressive and fearful behaviors in these cases [17]. Additionally, other factors that stand out are pain, fear due to late and inappropriate socialization, defense of territory or defense of their puppies, lack of social interaction with humans, predatory behavior [16], age, size and behavior of the victims and absence of other people in the surroundings [14]. Another factor that is generally included as an influence on the presentation of aggressiveness in dogs is the reproductive status of the animal (neutered or entire); however, there is still an important discussion in this regard, with multiple results found in the scientific literature, such as no effect on this behavior [18], decreased aggressiveness [19,20,21,22,23,24] or increased aggressiveness [25,26,27].

Fatal dog bite incidents are not only a public health problem, but can also be a challenge for the police investigation, as the victim’s injuries can be mistaken for signs of criminal aggression [17]. These cases are complex, since they require a multidisciplinary approach between veterinary pathologists, ethologists, forensic anthropologists and dentists who evaluate the scenario [28], where the dental analysis of the marks caused by the bite and traces of blood from the victim in the animal, for DNA analysis to determine the cause of death [17]. Likewise, it is important to take into account possible identification biases of the attacking dog’s guardian. This is due to the fact that the information is collected mainly from the victim of the incident, who avoided recognizing that the dog was their property.

In recent decades, a significant increase in the number of fatal attacks has been detected, as recorded in a study of 30 European countries over a 20-year period [11], a similar trend also observed in the United States [12]. Because of the above, it is essential to analyze the information related to this type of incidents in the scientific literature, in order to contribute to the understanding of the central problem, support decision making and improve the mechanisms for preventing canine bite events.

The present study aims to characterize the variables related to reports of death caused by dog bites in scientific articles during the years 2013–2017. For this, a systematic review of the indexed literature was conducted and variables were stratified into four pillars of analysis: characteristics of the victim, characteristics of the injuries and treatment of the victim, characteristics of the biting animal and its relationship with the victim and space-time scenario and associated factors.

## 2. Materials and Methods

### 2.1. Search Strategy

A systematic review of scientific articles on canine bites resulting in death, published between 2013 and 2017, was carried out at a global level. 

Literature searches were carried out using the databases: Academic Search Ultimate (EBSCO), CINAHL (EBSCO), Cochrane Library, MEDLINE (NLM), Scielo, Science Direct and Scopus.

The selected documentary sources were searched using standardized descriptors in search engines, where possible, in addition to strategies that included the keywords “dog” AND “bites” and “perro” AND “mordeduras”, and other derivatives in both English and Spanish, such as: “bitten” and “biting” and “mordeduras de perro”, among others. Based on the results found, we analyzed the incorporation of other keywords discovered in a first review of the same articles that could yield more relevant results, such as “dog aggression”, “wounds” and “attack”; and of certain exceptions in the search according to the origin of the source: for example, in Spanish, we added the word “canino” and “mordida”. Moreover, we truncated some search terms to find or eliminate variations of the same word, such as epidemi * and injur *.

### 2.2. Evidence Grading System Utilised in Systematic Review 

The systematic review of this study has a broad approach with the goal of capturing fatal dog bites across its spectrum of settings, worldwide.

We selected articles that met the inclusion criteria (*n* = 33), and this resulted in 12 articles (36.4%) that contained level 2 evidence and 21 articles (63.6%) that contained level 3 evidence. The data collection was searched, selected and extracted by three authors independently and discussed further in a focus group for inclusion in this review. To select the articles that were included in this review, the levels of evidence reported in the SIGN guidelines will be considered (Table 1). Only articles that had a level of evidence classification from 1 to 3 were included. In addition, the articles that were analyzed in the present work were categorized according to their risk of bias as low, medium and high, and in some cases where the information was insufficient to be classified at these levels, it was categorized as unclear risk, according to the SIGN criteria (Table 2).

### 2.3. Inclusion Criteria

All epidemiological studies and studies of injuries caused by canine bites resulting in death, published between 2013 and 2017, were included. This included primary and secondary articles, such as original research articles, reviews, case reports. Articles of all types of design and written in both Spanish and English were included.

### 2.4. Exclusion Criteria

In the databases that allowed it, exclusion criteria were applied in the search filters: years of publication was set to 2013 to 2017, and the type of documents was set to editorials, comments, letters, book reviews, etc., as they were not considered within the hierarchy of scientific evidence.

After analyzing the first results found, a series of key concepts were excluded by using the Boolean NOT in the search strategy:

NOT (“insect bites” OR “tick bites” OR “snake bites” OR “fly bites” OR “sand flies bites”).

**Figure 1:**
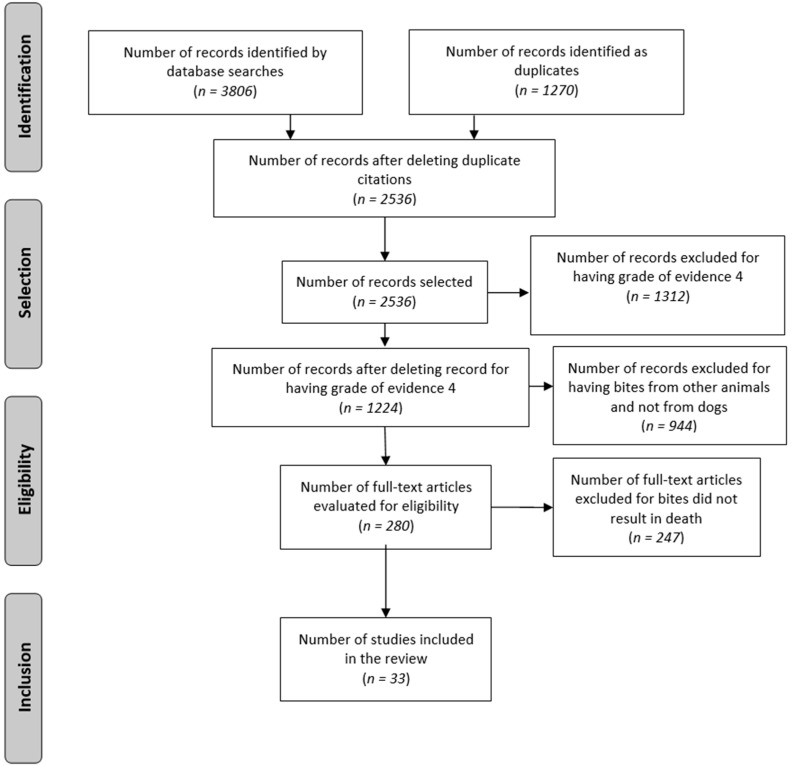
Flowchart of the information selection process from scientific articles to be analyzed in this study.

The present study considered four filtering stages: identification, selection, eligibility and inclusion. A total of 33 scientific articles with reports of deaths from canine bites were included (Figure 1).

### 2.5. Selected Variables

The following information was selected from each article, which was organized into the four groups of characteristics detailed below (Table 3).

### 2.6. Statistical Analysis

The information collected was stored and tabulated in Microsoft Excel 2016^®^ spreadsheets. To detect differences between two categories of a variable, the binomial test was used and if the variable had more than two categories, provided that the number of papers in which the variable was reported was greater than the number of unreported papers, the X2 homogeneity test was used. Variables with more than four categories and with a low number of reports were not statistically compared. GraphPad Prism 9.0 software was used for all statistical comparisons. Subsequently, a descriptive analysis was performed with the number of records found for all variables analyzed in this study. 

## 3. Results

Thirty-three scientific articles related to dog bite deaths were included, which contained different types of information for the different sections of the review.

### 3.1. Victim’s Characteristics

The number and proportion of incidents related to dog bite deaths according to sex, age group and occupation of the victim are shown in Table 4. A total of 57.6% of the victims were men (19/33) and the age group with the highest frequency of deaths was those aged 50 to 64 years (8/28).

### 3.2. Characteristics of the Injuries and Treatment of the Victim

The number and proportion of incidents related to dog bite deaths according to number of bites, treatment and anatomical area of the victim’s injury are shown in Table 5.

In the classification “number of bites”, the number of bites between single and multiple bites was similar (*p* > 0.05) and although it was not possible to compare body parts, it is observed that the upper limb represents 47.8% (11/23) of all bites.

Regarding the type of treatment received, washing, rabies and/or tetanus vaccine predominated at 59% (13/22), followed by antibiotic treatment at 27.3% (6/22).

### 3.3. Characteristics of the Biting Dog and Its Relationship with the Victim

The number and proportion of incidents related to dog bite deaths according to the victim–dog relationship, PDD and number of attacking dogs are shown in Table 6.

In 60% (12/20) of the selected articles, the biting dog was owned by the victim; a similar proportion was reported for whether the victim knew the biting animal (11/21).

Only 21.2% of the articles mentioned the dog’s breed (7/33). When these data were reported, PDD breeds involved in fatal incidents represented 66.7% (4/7).

In total, 60.6% (20/33) of the articles described a single attacking dog.

### 3.4. Spatiotemporal Scenario and Associated Factors

The number and proportion of incidents related to dog bite deaths according to the location of the attack, context, victim–dog approach and associated predisposing factors are shown in Table 7.

Only 12 articles mention the place of occurrence of the attack, among which the highest frequency was in public spaces—58.5% (7/12).

Only in four articles is the context of the attack mentioned (12.1%) (4/33): the dog approached the victim in three of them, and the human approached the dog in one.

The medical factors of the victim that are associated with death are described in 81.8% (27/33) of the articles, and the lack of post-exposure prophylaxis or incomplete prophylaxis are highlighted in 51.9% (14/27) of these papers.

## 4. Discussion

The total number of articles with records of incidents of dog bites with a fatal outcome was 33, a number that may be limited to obtain definitive conclusions; however, it allows describing a general scenario regarding the characteristics of the victims, injuries and treatments carried out and, to a lesser extent, the characteristics of the aggressor animals and some background information on the context in which they occur.

### 4.1. Victim’s Characteristics

The sex of the deceased was mentioned in 100% (33/33) of the articles. In 57.6% (19/33) of the articles, the sex of the victim was identified as male. Examples of these investigations are those carried out in the US [7,12,19,29], Canada [14] and Spain [30,31]. These results are consistent with those found in Europe by Sarenbo and Svensson [11], where fatal attacks occur more frequently in men of all age groups, with the exception of those over 80 years of age [11]. This is a commonly recorded epidemiological variable, so it is possible to incorporate it into research. There are behavioral and cultural aspects that explain why men are more frequent victims of these aggressions and also suffer more severe injuries, which can lead to death, such as risky behavior in front of dogs, violent games or physical punishment [21], trades such as postmen, readers of basic services or preferences for certain types of breeds [22,23].

It was not possible to statistically compare the different age strata, due to the low number of reports in several of them; however, the participation of adults is observed more frequently (stratum 7) in this type of report (8/28). In the study carried out by Mora et al. [30] in Spain, the highest number of deaths caused by canine bites (70.6%) occurred in people over 50 years of age. This contrasts with the results obtained in other studies where it is reported that children have a greater participation in fatal dog bite incidents; for example, the findings registered in Canada reported that 85.7% of the victims were children under 12 years [14], while another study from New Zealand reported that 39% were children under 10 years [32], an investigation carried out in the US reported that 55.6% of the fatal victims were children under 10 years [12] and finally another study from the US reported that 45.3% of the victims were children under 5 years [7]. However, a more recent study [11], using data from 30 European countries over 20 years, showed a bimodal distribution of deaths in different age categories, with a first peak at ages 1–9 years, coinciding with what was stated previously [12,17], and with an increase in deaths after 40 years, more similar to our results and what was stated by Mora et al. [16]. This contrast in age category reflects the significant variability between age strata involved in incidents of canine bites resulting in death, which could also be explained by the different forms of age registration and classifications in the original data sources or those carried out by the authors for the purposes of their studies.

The highest report of deaths in people over 50 years of age can be explained by various comorbidities presented by this age group, which could in some cases immunocompromise the victim, leaving them more vulnerable to sepsis caused by bacteria from the dog’s oral microbiota. This coincides with the results obtained in the present review, where the percentage of immunocompromised fatalities belonged to stratum 7 (14.8%) (4/27). These bacteria would mainly be *Capnocytophaga canimorsus*, which manages to proliferate more easily in splenectomized patients (35%), with alcohol abuse (35%) or immunocompromised patients by corticosteroid treatments, hematological neoplasms or autoimmune diseases (17%). Other bacteria mentioned in various studies were *Streptococcus canis*, *Pasteurella multocida* and *Prevotella* spp. [3,6,33,34]. These findings are in contrast to those found by Sarenbo and Svensson [11], since they found that both young children and older adults have higher mortality, mainly due to the vulnerability of the victim to die from injuries caused by bites (young children) or blows caused by the attack of the dog (older adults). That is, in our study, the mobility of people had an important impact on the outcome of death, more than the trauma or exsanguination of the victims.

Other predisposing factors for this age stratum could be reduced mobility or physical or mental disability (cognitive impairment), which prevent the person from being able to defend themselves or ask for help during the attack [7,8,9]. Furthermore, a study carried out in the US reported that in 87.1% of the cases, no person without disabilities was close enough to the victim to intervene [7]. In the latter case, the attacked person died of hypovolemic shock due to the severity of the injuries [30]. 

### 4.2. Characteristics of the Injuries and Treatment of the Victim

When evaluating injury information for deceased victims, a higher proportion of records mentioning single injuries was observed (e.g., Stratum 7 (57.14%) (4/7)). In the United States [29] and Spain [30], the highest proportion of single injuries was recorded in adults. Multiple injuries are more common in children [29,30] due to their reduced ability to defend themselves or flee once the first injury occurs.

The most affected anatomical area was the upper extremities (11/23), followed by the head and neck (6/23). It is expected that head and neck injuries have a greater prevalence in fatal cases, as the victim would be more likely to die from vascular trauma, air embolism due to damage to the veins in the neck and/or closed head trauma due to crushing of the skull. Crushing neck trauma can cause carotid artery dissection, cervical cord injury or asphyxia [35,36]. Injuries to the upper extremities are associated with defensive injuries [32].

In the present review, in the youngest strata (Stratum 1, 2 and 3) 66.66% (4/6) of the victims were bitten on the head and neck. It should be noted that head and neck injuries are more frequent in children, due to their short stature and the proximity of their head to the dog’s jaw [30,37,38]. By contrast, upper limb injuries occur more frequently in people older than 15 years [32], possibly due to defensive acts.

### 4.3. Characteristics of the Biting Animal and Its Relationship with the Victim

In the victim–dog relationship variable, half of the reports included people who knew the biting animal (11/21). Examples of these studies are those carried out in Spain [16] and the United States [29], which report that there are no differences in whether the victim did or did not know the dog. Other studies reported more frequent cases where the biting dog knew the victim (57.1%).

The low number of articles that mention the breed of the biting animal can be explained by the lack of registration of these data in the healthcare centers, where it is not recorded due to ignorance of the relevance of this antecedent or because the affected person is not able to inform the center of the breed. It should be noted that, although the records are few, most of the studies report animals of PDD due to biases related to regulations that require reporting this antecedent, intentional search for attacks by these animals and biases in the classification, among others. It is for this reason that some authors point out that caution should be exercised in the analysis of the participation of PDD breeds, since these could be overrepresented due to the fact that there is a greater proportion of individuals of these breeds or their crosses in certain countries, such as, for example, the German Shepherd in New Zealand [24]. In addition, the identification of the breed of the biting animal is carried out visually and mostly by people that are unspecialized in cynology, which is, thus, unreliable [39].

Among the number of attacking animals, the attack of a single animal was reported more frequently (16/20), which coincides with studies carried out previously, where it has been stated that attacks produced by packs of dogs are extremely rare and only constitute 6.9% of all fatal dog attacks [40,41].

### 4.4. Spatiotemporal Scenario and Associated Factors

Of places where the mortal attack takes place, the public space is the most frequent at 58.5% (7/12). This result coincides with that obtained by Healey et al. [24], who reported that only 6% of the attacks occurred on public roads.

Of the victim’s medical factors associated with death, people who did not receive post-exposure rabies prophylaxis or who received incomplete treatment were predominant, i.e., 51.9% (14/27). Rabies continues to be a priority public health problem in countries with poor prevention and control programs [42,43]. In the reviewed publications, many victims suffered from splenectomy. This condition could make them more vulnerable to being bitten by negatively impacting their immunity and ability to survive a bacterial infection [44,45].

Mental illnesses and/or disabilities was in 18.5% of cases (5/27) and alcoholism in 11.1% of cases (3/27); these conditions would not allow the victims to interact adequately with the dogs [7] and could provoke the initiation of the fatal attack.

The aforementioned reasons show the complexity of the multiple characteristics that can influence these types of incidents, and the importance of reviews of this type is evident in order to facilitate a comprehensive understanding of said problem.

### 4.5. Study Limitations

Among the limitations of the study is the low number of published reports for the variables that are relevant. This scenario complicates the understanding of the problem; however, it is the reality that currently exists and the analysis, considering potential biases, allows us to understand the type of information that is reaching the community to inform public policy decisions and develop prevention programs.

Since the existing biases in the articles incorporated in this review cannot be eliminated, they have been declared and classified, so that the reader can consider the existing level of them and come to an informed conclusion after analyzing the antecedents from this work.

The data collection methods used to produce this type of article still require greater detail and standardization in order to facilitate the analysis of the literature associated with dog bites. Additionally, limitations in identifying breeds involved in fatalities may lead to confusion in the analysis of the animals involved in such incidents. It would be ideal to have methods of genetic determination of the breed involved; however, it is also very useful to be able to analyze the information from visual identification of the offending animal, in order to get closer to the scene of the incident.

Despite the limitations mentioned above, this review incorporates the most relevant variables for dealing with this type of incident, using the information that is currently available in the scientific literature. Therefore, in the discussion of this work, the information has been correctly analyzed, considering all these possible biases.

## 5. Conclusions

Bites, especially dog bites, constitute a problem and interest in public health, due to their frequency as well as the complications that they can cause, including the death of the affected persons. Although a fatal outcome is a rare situation, there are ethical, health and social implications that must be analyzed in order to understand the related factors and define strategies to minimize the problem, considering that they are preventable events, with greater attention to the fatal outcomes.

Most of the scientific publications have information on the affected people, with the most common variables in all the works being age, sex and country of residence of those affected, and the less common variables being the affected anatomical area, number of injuries, the treatments carried out and the conditions that could increase the probability of fatal outcome.

The information related to the characteristics of the aggressor animal and the contexts in which the incidents occur are less often reported. Systems of information records on bite accidents should be implemented in healthcare services, as well as promoting studies on human–animal interactions and the context of bites, in order to have objective and sufficient antecedents to define strategies and implement plans and programs that allow to minimize bite incidents, with special emphasis on those with a fatal outcome. Finally, it is important to consider the multiple biases that the analyzed literature may have, be it in methodology, sample number, etc. This is to avoid oversizing some results, which can lead to an error in making decisions to address this problem.

## Figures and Tables

**Table 1:** The hierarchy of evidence utilized in this systematic review, amended from SIGN.

Level	Description of Studies Meeting This Level
1++	High quality meta-analyses, systematic reviews of RCTs or RCTs with a very low risk of bias.
1+	Well-conducted meta-analyses, systematic reviews or RCTs with a low risk of bias.
1−	Meta-analyses, systematic reviews or RCTs with a high risk of bias.
2++	High quality case control, cohort and cross-sectional studies with a very low risk of confounding or bias and a high probability that the relationship is causal.
2+	Well-conducted case control, cohort or analytical cross-sectional studies with a low risk of confounding or bias and a moderate probability that the relationship is causal.
2−	Case control, cohort or analytical cross-sectional studies with a high risk of confounding or bias and a significant risk that the relationship is not causal.
3	Non-analytic studies, e.g., case reports, case series and descriptive cross-sectional studies.
4	Expert opinion.

**Table 2:** Studies reaching the inclusion standard of the systematic review.

	Article Name	Study Type	Study Population Size	Study Location	Levels of Evidence	Risk of Bias
1	Awareness of rabies and response to dog bites in a Bangladesh community	Cross-sectional	3200 people	Bangladesh	2++	Low risk
2	Co-occurrence of potentially preventable factors in 256 dog bite-related fatalities in the United States (2000–2009)	Cohort study	256 people	USA	2++	Low risk
3	Establishment of a Canine Rabies Burden in Haiti through the Implementation of a Novel Surveillance Program	Cohort study	1003 people	Haiti	2++	Low risk
4	Time series analysis and mortality model of dog bite victims presented for treatment at a referral clinic for rabies exposure in Monrovia, Liberia, 2010–2013	Retrospective cohort study	775 people	Liberia	2++	Low risk
5	Use of statewide emergency department surveillance data to assess incidence of animal bite injuries among humans in North Carolina	Retrospective Cohort AND cross-sectional study	29586 people	USA	2++	Low risk
6	Characteristics of 1616 Consecutive Dog Bite Injuries at a Single Institution	Cohort study	1616 people	USA	2+	Low risk
7	Dog bite injuries in children: Clinical implications for head involvement	Cohort study	236 people	USA	2+	High risk
8	Bites from the same dog, different outcomes for two patients: a case report	Case Study	2 people	China	2−	Low risk
9	Dog Bite Health Burden in Alaskan Communities 2002-12	Cross-sectional	292 people	USA	2−	High risk
10	Human rabies: A descriptive observation of 21 children in Kinshasa, The democratic republic of Congo	Retrospective cohort study	21 people	Democratic Republic of Congo	2−	High risk
11	Intracranial Injuries from Dog Bites in Children	Retrospective cohort study	10 people	USA	2−	High risk
12	Severe Penile Injuries in Children and Adolescents: Reconstruction Modalities and Outcomes	Retrospective cohort study	2 people	Serbia	2−	High risk
13	A confirmed rabies case in a French resident in Cambodia, June 2015	Case report	1 person	Cambodia	3	Low risk
14	A fatal case of hypothermia caused by dog bites	Case report	1 person	China	3	High risk
15	A rare case of Waterhouse-Friderichsen syndrome caused by Capnocytophaga canimorsus in an immunocompetent patient	Case report	1 person	USA	3	Unclear risk
16	Acute flaccid paralysis following spinal anaesthesia: A diagnostic dilemma	Case report	1 person	Sri Lanka	3	Low risk
17	An Unusual Case of Predation: Dog Pack or Cougar Attack?	Case report	1 person	Argentina	3	Unclear risk
18	Bigger than his bite	Case report	1 person	USA	3	Unclear risk
19	Capnocytophaga canimorsus sepsis in a methotrexate-treated patient with rheumatoid arthritis	Case report	1 person	Japan	3	Unclear risk
20	Case 10-2014: A 45-year-old man with a rash	Case report	1 person	USA	3	Unclear risk
21	Case of toxic epidermal necrolysis following a dog bite	Case report	1 person	Australia	3	High risk
22	Diagnosis, management and post-mortem findings of a human case of rabies imported into the United Kingdom from India: A case report	Case report	1 person	U.K.	3	Low risk
23	Dog-mediated human rabies death, Haiti, 2016	Case report	1 person	Haiti	3	Low risk
24	Domestic Predation of an Elder: A Fatal Dog Attack Case	Case report	1 person	France	3	Low risk
25	Man’s best friend, fatal in the end	Case report	1 person	USA	3	Unclear risk
26	Management of severe musculoskeletal trauma following a dog mauling attack in a nonagenarian	Case report	1 person	USA	3	Unclear risk
27	Multidisciplinary approach to fatal dog attacks: A forensic case study	Case report	1 person	Tunisia	3	Low risk
28	Rabies encephalitis in a child: A failure of rabies post exposure prophylaxis?	Case report	1 person	Tunisia	3	Low risk
29	Rabies encephalitis with an unusually long latency period	Case report	1 person	India	3	Unclear risk
30	Same dog bite and different outcome in two cases-Case report	Case report	2 people	India	3	Unclear risk
31	Spontaneous pneumomediastinum due to paralytic rabies	Case report	1 person	China	3	Unclear risk
32	Survival of a newborn from a pregnant woman with rabies infection	Case report	1 person	China	3	Unclear risk
33	The use of genetic markers to estimate relationships between dogs in the course of criminal investigations	Case report	1 person	Italy	3	Low risk

**Table 3:** Classification of the variables of interest selected for the study.

Variable	Variable Description	Variable Classification
**Characteristics of the Victim**		
Sex	Biological sex of the bitten personBoth means that men and women were present.	Man
		Woman
		Both
		Not reported
Age stratum	Age group of the victim, measured in years	Stratum 1 (0–4 years)
		Stratum 2 (>4–9 years)
		Stratum 3 (>9–14 years)
		Stratum 4 (>14–25 years)
		Stratum 5 (>25–35 years)
		Stratum 6 (>35–49 years)
		Stratum 7 (>49–64 years)
		Stratum 8 (≥65 years)
		Not reported
Occupation of the victim	Reported occupation of the dog bite victim	Homemaker
		Jobless
		Dependent worker
		Independent worker
		Tourist
		Not reported
Residence of the victim	Classification by type of residence of the victim (permanent or temporary residence)	Resident
		Tourist
		Not reported
**Characteristics of the Injuries and Treatment**		
Number of bites	No. of reported bites	Single
		Multiple
		Not reported
Treatment type	Clinical, pharmacological, surgical or other interventions applied to the bitten person	Washing, rabies shot, tetanus shot
		Antibiotic
		Surgery
		No treatment
		Not reported
Anatomical area of the injury	Place of the body where the injury caused by the biting animal is located.	Head and neck
		Upper extremity
		Lower extremity
		Another single area
		Multiple areas
		Not reported
**Characteristics of the Biting Dog and Its Relationship with the Victim**		
Report of the relationship between victim and biting dog	Status of possession of the dog by a responsible person, guardian or owner	Dog belonged to the victim
		Dog did not belong to the victim
		Not reported
Potentially dangerous dog (PDD)	Dog belonging to a breed, or its crosses, with potential aggressive characteristics in accordance with the regulations of each country or territory	Reports with PDD breeds (hybrid doberman/rottweiler
		german shepherd
		pit bull
		staffordshire bull terrier, not reported)
		Reports with no PDD breeds (labrador retriever, boston terrier, mongrel, not reported)
		Not reported
Victim’s familiarity with the biting dog	Statement of knowledge of the owner, address or habitual location of the biting dog	Known animal
		Unknown animal
		Not reported
Number of attacking dogs	Total number of dogs participating in the fatal attack	Multiple dogs
		One dog
		Not reported
**Spatiotemporal Scenario and Associated Factors**		
Country of study	Author’s Country of Origin	Argentina
		Australia
		Bangladesh
		Cambodia
		China
		France
		Haiti
		India
		Italy
		Japan
		Nigeria
		United Kingdom
		Democratic Republic of Congo
		Serbia
		Sri Lanka
		Tunisia
		United States
		Not reported
Attack location	Site where the bite incident occurred	Inside the dog house
		Inside the victim’s house
		Public space
		Not reported
Attack context	Situation or interaction between the affected person and the biting animal in which the biting incident occurred	Eating
		Fighting with another dog
		Person walking or running
		Other
		Not reported
Type of approach in the attack	Circumstance in which the incident occurred, regarding the approach between the victim and the dog	Human to dog
		Dog to human
		Not reported
Medical factors of the victim	Medical condition of the victim associated with death	No or incomplete post-exposure prophylaxis
		Splenectomized patient Immunocompromised patient
		Alcoholism
		Mental illness, disability
		Not reported
Cause of death	Pathophysiological mechanisms triggering a fatal outcome	Bacterial infection (Capnocytophaga canimorsus, not reported)
		Viral infection (Rabies)
		Hypovolemic shock
		Hypothermia
		Not reported

**Table 4:** Characteristics of the victim, collected from 33 articles on dog bites resulting in death.

Article Variables	*n* (%)
Sex of the victim	
Male	19 (57.6%) ^a^
Female	11 (33.3%) ^b^
Both	3 (9.1%) ^c^
Reported	33 (100%)
Not reported	0 (0%)
Age stratum of the victim	
Stratum 1 (0–4 years)	4 (14.3%)
Stratum 2 (>4–9 years)	1 (3.5%)
Stratum 3 (>9–14 years)	1 (3.5%)
Stratum 4 (>14–25 years)	4 (14.3%)
Stratum 5 (>25–35 years)	4 (14.3%)
Stratum 6 (>35–49 years)	3 (10.7%)
Stratum 7 (>49–64 years)	8 (28.6%)
Stratum 8 (≥65years)	3 (10.7%)
Reported	28 (82.1%)
Not reported	5 (17.9%)
Occupation of the victim	
Homemaker	1 (20%)
Unemployed	1 (20%)
Dependent worker	2 (40%)
Self-employed	1 (20%)
Reported	5 (17.9%)
Not reported	28 (82.1%)
Residence of the victim	
Resident	31 (93.9%) ^a^
Tourist	2 (6.1) ^b^
Reported	33 (100%)
Not reported	0 (0%)

Subscripts with different letters indicate significant differences *p* < 0.05 between the proportions.

**Table 5:** Characteristics of the injuries and treatment of the victim collected from 33 articles on dog bites resulting in death.

Article Variables	*n* (%)
Number of bites	
Single	15 (62.5%) ^a^
Multiple	9 (37.5%) ^a^
Reported	24 (72.7%)
Not reported	9 (27.3%)
Treatment type	
Washing, rabies shot and tetanus shot	13 (59%) ^a^
Antibiotic	6 (27.3%) ^b^
Surgery	2 (9.1%) ^b,c^
No treatment	1 (4.6%) ^c^
Reported	22 (66.7%)
Not reported	11 (33.3%)
Anatomical area of the injury	
Head, neck	6 (26.1%) ^a,b^
Upper extremity	11 (47.8%) ^a^
Lower extremity	3 (13%) ^b,c^
Another single area	1 (4.4%) ^c^
Multiple areas	2 (8.7%) ^b,c^
Reported	23 (69.7%)
Not reported	10 (30.3%)

Subscripts with different letters indicate significant differences *p* < 0.05 between the proportions.

**Table 6:** Information on the biting dog and its relationship with the victim, collected from 33 articles on dog bites resulting in death.

Article Variable	*n* (%)
Victim–dog ownership relationship	
Dog belonged to the victim	12 (60%) ^a^
Dog did not belong to the victim	8 (40%) ^a^
Reported	20 (60.6%)
Not reported	13 (39.4%)
Victim–dog relationship	
Known dog	11 (52.4%) ^a^
Unknown dog	10 (47.6%) ^a^
Reported	21 (63.6%)
Not reported	12 (36.4%)
PDD	
PDD breed	4 (66.7%)
Doberman/Rottweiler Hybrid	1 (25%)
Pit bull	2 (50%)
Staffordshire Bull Terrier	1 (25%)
Not reported	0 (0%)
No PDD breed	3 (33.3%)
Labrador retriever	1 (33.3%)
Boston terrier	1 (33.3%)
Mongrel	1 (33.3%)
Not reported	0 (0%)
Reported Not reported	7 (21.2%) 26 (78.8%)
Number of attacking dogs	
Multiple dogs	4 (20%) ^a^
One dog	16 (80%) ^b^
Reported	20 (60.6%)
Not reported	13 (39.4)

Subscripts with different letters indicate significant differences *p* < 0.05 between the proportions.

**Table 7:** Information on the spatiotemporal scenario and associated factors, collected from 33 articles on dog bites resulting in death.

Article Variable	*n* (%)
Country of the study	
Argentina	1 (3%)
Australia	1 (3%)
Bangladesh	1 (3%)
Cambodia	1 (3%)
China	4 (12.3%)
France	1 (3%)
Haiti	1 (3%)
India	2 (6%)
Italy	1 (3%)
Japan	1 (3%)
Nigeria	1 (3%)
United Kingdom	1 (3%)
Democratic Republic of Congo	1 (3%)
Serbia	1 (3%)
Sri Lanka	1 (3%)
Tunisia	2 (6%)
United States	12 (36.6%)
Reported	33 (100%)
Not reported	0 (0%)
Location of the attack	
Inside the dog house	5 (41.7%)
Inside the victim’s house	0 (0%)
Public space	7 (58.5%)
Reported	12 (36.4%)
Not reported	21 (63.6%)
Context of the attack	
Eating	1 (14.3%)
Fighting with another dog	1 (14.3%)
Person walking or running	2 (28.6%)
Other	3 (42.8%)
Reported	7 (21.2%)
Not reported	26 (78.8%)
Type of approach in the attack	
Human to dog	1 (25%)
Dog to human	3 (75%)
Reported	4 (12.1%)
Not reported	29 (87.9%)
Medical factors of the victim.	
No or incomplete post-exposure prophylaxis	14 (51.9%) ^a^
Splenectomized patient	1 (3.7%) ^b^
Immunocompromised patient	4 (14.8%) ^b^
Alcoholism	3 (11.1%) ^b^
Mental illness, disability	5 (18.5%) ^b^
Reported	27 (81.8%)
Not reported	6 (18.2%)
Mechanism of death	
Bacterial infection	7 (23.3%) ^a^
Capnocytophaga canimorsus	6 (85.7)
Not reported	1(14.3%)
Viral infection (Rabies)	14 (46.7%) ^a^
Hypovolemic shock	8 (26.7%) ^a^
Hypothermia	1 (3.3%) ^b^
Reported	30 (90.9%)
Not reported	3 (9.1%)

Subscripts with different letters indicate significant differences *p* < 0.05 between the proportions.

## References

[1] Organización Mundial de la Salud (2018). Mordeduras de Animales. https://www.who.int/es/news-room/fact-sheets/detail/animal-bites.

[2] Damborg P., Broens E.M., Chomel B.B., Guenther S., Pasmans F., Wagenaar J.A., Weese J.S., Wieler L.H., Windahl U., Vanrompay D. (2016). Bacterial Zoonoses Transmitted by Household Pets: State-of-the-Art and Future Perspectives for Targeted Research and Policy Actions. J. Comp. Pathol..

[3] Taniyama D., Abe Y., Sakai T., Kikuchi T., Takahashi T. (2017). Human case of bacteremia caused by Streptococcus canis sequence type 9 harboring the scm gene. IDCases.

[4] Delman M., Chalikonda D., Haroian N., Djurkovic S. (2017). Capnocytophaga canimorsus meningitis in an immunocompetent woman: A case report and review of the literature. Infect. Dis. Clin. Pract..

[5] Audu S.W., Mshelbwala P.P., Jahun B.M., Bouaddi K., Weese J.S. (2019). Two fatal cases of rabies in humans who did not receive rabies postexposure prophylaxis in Nigeria. Clin. Case Rep..

[6] Butler T. (2015). Capnocytophaga canimorsus: An emerging cause of sepsis, meningitis, and post-splenectomy infection after dog bites. Eur. J. Clin. Microbiol. Infect. Dis..

[7] Patronek G.J., Sacks J.J., Delise K.M., Cleary D.V., Marder A.R. (2013). Co-occurrence of potentially preventable factors in 256 dog bite-related fatalities in the United States (2000–2009). J. Am. Vet. Med. Assoc..

[8] Li Y., Shen R., Ding R., Wen G., Du A., Dong Z., Ren X., Yao H., Zhu B., Li R. (2017). A fatal case of hypothermia caused by dog bites. Med. Sci. Law.

[9] Le Garff E., Mesli V., Delannoy Y., Pollard J., Becart A., Hedouin V. (2017). Domestic Predation of an Elder: A Fatal Dog Attack Case. J. Forensic Sci..

[10] World Health Organisation (2011). Weekly epidemiological record. PLoS Negl. Trop. Dis..

[11] Sarenbo S., Svensson P.A. (2021). Bitten or struck by dog: A rising number of fatalities in Europe, 1995–2016. Forensic Sci. Int..

[12] Langley R.L. (2009). Human fatalities resulting from dog attacks in the united states, 1979–2005. Wilderness Environ. Med..

[13] Heinze S., Feddersen-Petersen D.U., Tsokos M., Buschmann C., Püschel K. (2014). Tödliche Attacken von Hunden auf Kinder: Aktualgenese und Motivation bei spezifischer Kasuistik und bestimmten pathomorphologischen Veränderungen. Rechtsmedizin.

[14] Raghavan M. (2008). Fatal dog attacks in Canada, 1990–2007. Can. Vet. J..

[15] Våge J., Bønsdorff T.B., Arnet E., Tverdal A., Lingaas F. (2010). Differential gene expression in brain tissues of aggressive and non-aggressive dogs. BMC Vet. Res..

[16] Van Den Berg L. (2016). Genetics of dog behavior. The Domestic Dog: Its Evolution, Behavior and Interactions with People.

[17] Arvelius P., Eken Asp H., Fikse W.F., Strandberg E., Nilsson K. (2014). Genetic analysis of a temperament test as a tool to select against everyday life fearfulness in rough collie. J. Anim. Sci..

[18] Palestrini C., Mazzola S.M., Caione B., Groppetti D., Pecile A.M., Minero M., Cannas S. (2021). Influence of gonadectomy on canine behavior. Animals.

[19] Hopkins S.G., Schubert T.A., Hart B.L. (1976). Castration of adult male dogs: Effects on roaming, aggression, urine marking, and mounting. J. Am. Vet. Med. Assoc..

[20] Hart B.L. (1976). Behavioral effects of castration. Canine Pract..

[21] Wright J.C., Nesselrote M.S. (1987). Classification of behavior problems in dogs: Distributions of age, breed, sex and reproductive status. Appl. Anim. Behav. Sci..

[22] Knol B.W., Egberink-Alink S.T. (1989). Treatment of problem behaviour in dogs and cats by castration and progestagen administration: A review. Vet. Q..

[23] Maarschalkerweerd R.J., Endenburg N., Kirpensteijn J., Knol B.W. (1997). Influence of orchiectomy on canine behaviour. Vet. Rec..

[24] Neilson J.C., Eckstein R.A., Hart B.L. (1997). Effects of castration on problem behaviors in male dogs with reference to age and duration of behavior. J. Am. Vet. Med. Assoc..

[25] Hart B.L., Eckstein R.A. (1997). The role of gonadal hormones in the occurrence of objectionable behaviours in dogs and cats. Appl. Anim. Behav. Sci..

[26] Bennett P.C., Rohlf V.I. (2007). Owner-companion dog interactions: Relationships between demographic variables, potentially problematic behaviours, training engagement and shared activities. Appl. Anim. Behav. Sci..

[27] Farhoody P., Mallawaarachchi I., Tarwater P.M., Serpell J.A., Duffy D.L., Zink C. (2018). Aggression toward familiar people, strangers, and conspecifics in gonadectomized and intact dogs. Front. Vet. Sci..

[28] Salem N.H., Belhadj M., Aissaoui A., Mesrati M.A., Chadly A. (2013). Multidisciplinary approach to fatal dog attacks: A forensic case study. J. Forensic Leg. Med..

[29] Shields L.B.E., Bernstein M.L., Hunsaker J.C., Stewart D.M. (2009). Dog bite-related fatalities: A 15-year review of Kentucky medical examiner cases. Am. J. Forensic Med. Pathol..

[30] Mora E., Fonseca G.M., Navarro P., Castaño A., Lucena J. (2018). Fatal dog attacks in Spain under a breed-specific legislation: A ten-year retrospective study. J. Vet. Behav..

[31] Rosado B., García-Belenguer S., León M., Palacio J. (2009). A comprehensive study of dog bites in Spain, 1995–2004. Vet. J..

[32] Healey D. (2007). Fatal dog bites in New Zealand. N. Z. Med. J..

[33] Westgarth C., Brooke M., Christley R.M. (2018). How many people have been bitten by dogs? A cross-sectional survey of prevalence, incidence and factors associated with dog bites in a UK community. J. Epidemiol. Community Health.

[34] Wright J.C. (1991). Canine aggression toward people. Bite scenarios and prevention. Vet. Clin. N. Am. Small Anim. Pract..

[35] Giordano A., Dincman T., Clyburn B.E., Steed L.L., Rockey D.C. (2015). Clinical features and outcomes of Pasteurella multocida infection. Medicine.

[36] Jacob J., Lorber B. (2015). Diseases transmitted by man’s best friend: The dog. Microbiol. Spectr..

[37] Calkins C.M., Bensard D.D., Partrick D.A., Karrer F.M. (2001). Life-threatening dog attacks: A devastating combination of penetrating and blunt injuries. J. Pediatr. Surg..

[38] Lauridson J.R., Myers L. (1993). Evaluation of Fatal Dog Bites: The View of the Medical Examiner and Animal Behaviorist. J. Forensic Sci..

[39] Bernardo L.M., Gardner M.J., Rosenfield R.L., Cohen B., Pitetti R., O’Neill K.A. (2002). A comparison of dog bite injuries in younger and older children treated in a pediatric emergency department. Pediatr. Emerg. Care.

[40] De Keuster T., Lamoureux J., Kahn A. (2006). Epidemiology of dog bites: A Belgian experience of canine behaviour and public health concerns. Vet. J..

[41] Voith V.L., Trevejo R., Dowling-Guyer S., Chadik C., Marder A., Johnson V., Irizarry K. (2013). Comparison of Visual and DNA Breed Identification of Dogs and Inter-Observer Reliability. Am. J. Sociol. Res..

[42] Sacks J.J., Lockwood R., Hornreich J., Sattin R.W. (1996). Fatal dog attacks, 1989–1994. Pediatrics.

[43] Avis S.P. (1999). Dog pack attack: Hunting humans. Am. J. Forensic Med. Pathol..

[44] Eke C., Omotowo I., Ukoha O., Ibe B. (2015). Human rabies: Still a neglected preventable disease in Nigeria. Niger. J. Clin. Pract..

[45] Huang X.-Y., Li X.-L., Wu S.-Y., Gu Y.-L., Lv X.-J., Klena J.D., Xu B.-L. (2017). Bites from the same dog, different outcomes for two patients: A case report. Infect. Dis. Poverty.

